# Virally-vectored vaccine candidates against white-nose syndrome induce anti-fungal immune response in little brown bats (*Myotis lucifugus*)

**DOI:** 10.1038/s41598-019-43210-w

**Published:** 2019-05-01

**Authors:** Tonie E. Rocke, Brock Kingstad-Bakke, Marcel Wüthrich, Ben Stading, Rachel C. Abbott, Marcos Isidoro-Ayza, Hannah E. Dobson, Lucas dos Santos Dias, Kevin Galles, Julia S. Lankton, Elizabeth A. Falendysz, Jeffrey M. Lorch, J. Scott Fites, Jaime Lopera-Madrid, J. Paul White, Bruce Klein, Jorge E. Osorio

**Affiliations:** 1 US Geological Survey, National Wildlife Health Center, Madison, Wisconsin USA; 2 Department of Pathobiological Sciences, School of Veterinary Medicine, University of Wisconsin - Madison, Madison, Wisconsin USA; 3 Departments of Pediatrics, School of Medicine and Public Health, University of Wisconsin - Madison, Madison, Wisconsin USA; 4 Internal Medicine, School of Medicine and Public Health, University of Wisconsin - Madison, Madison, Wisconsin USA; 5 Microbiology and Immunology, School of Medicine and Public Health, University of Wisconsin - Madison, Madison, Wisconsin USA; 6 Wisconsin Department of Natural Resources, Madison, Wisconsin USA

**Keywords:** Immunology, Fungal infection

## Abstract

White-nose syndrome (WNS) caused by the fungus, *Pseudogymnoascus destructans* (*Pd*) has killed millions of North American hibernating bats. Currently, methods to prevent the disease are limited. We conducted two trials to assess potential WNS vaccine candidates in wild-caught *Myotis lucifugus*. In a pilot study, we immunized bats with one of four vaccine treatments or phosphate-buffered saline (PBS) as a control and challenged them with *Pd* upon transfer into hibernation chambers. Bats in one vaccine-treated group, that received raccoon poxviruses (RCN) expressing *Pd* calnexin (CAL) and serine protease (SP), developed WNS at a lower rate (1/10) than other treatments combined (14/23), although samples sizes were small. The results of a second similar trial provided additional support for this observation. Bats vaccinated orally or by injection with RCN-CAL and RCN-SP survived *Pd* challenge at a significantly higher rate (P = 0.01) than controls. Using RT-PCR and flow cytometry, combined with fluorescent *in situ* hybridization, we determined that expression of IFN-γ transcripts and the number of CD4 + T-helper cells transcribing this gene were elevated (P < 0.10) in stimulated lymphocytes from surviving vaccinees (n = 15) compared to controls (n = 3). We conclude that vaccination with virally-vectored *Pd* antigens induced antifungal immunity that could potentially protect bats against WNS.

## Introduction

Since its discovery in New York in 2006^1^, white-nose syndrome (WNS), caused by the fungus *Pseudogymnoascus (Geomyces*) *destructans* (*Pd*), has killed at least 7 million bats in the US^2^, causing significant population declines in numerous bat species. The once ubiquitous little brown bat (*Myotis lucifugus*) is particularly sensitive and has experienced severe population declines in many states^3^. This emerging disease may also have important economic implications as bats consume many crop and forests pests, providing key ecological services to US agriculture^4^. The fungus, which only infects bats during hibernation, is spreading across the US at an alarming rate, having reached Midwestern states by the winter of 2010 and Washington state in the winter of 2016^5^.

Current proposed methods for control of WNS in bats include application of plant – derived compounds or bacteria to hibernating bats to directly inhibit the growth of the fungus^6–10^ or inhibition by bacterially produced volatile organic compounds^11^. While results from these studies show promise in the laboratory for reducing the growth of *Pd*, their practical application may be hindered by logistical factors such as the amount of agent needed or unintended consequences such as the disruption of skin flora or cave ecosystems. Furthermore, most proposed methods would require treatment of bats in the winter, and treatment application when bats are hibernating may be problematic or impractical for some species.

Vaccination may offer an alternative method of disease control. Historically, few fungal vaccines have been developed and used, but the increasing prevalence and severity of invasive fungal pathogens in both humans and animals has led to expanded research and many promising developments in this area^12^. For managing WNS in bats, practical delivery of an oral vaccine could be achieved through topical application via liquid gels or pastes that are ingested by bats during grooming; it could be applied months prior to hibernation, without requiring access to, and disruption of, cave ecosystems. Additionally, vaccination may generate long-lasting immunity without requiring repeated annual treatments. Delivering oral vaccines to wildlife has been successfully achieved through the use of poxviral vectors^13,14^ such as vaccinia (e.g. oral rabies vaccine for carnivores) and raccoon poxvirus (RCN; e.g. sylvatic plague vaccine for prairie dogs). Our previous studies have shown that attenuated RCN is a suitable vaccine vector for bats as it was able to infect *Tadarida brasiliensis* for a limited time period when given via the oronasal route and produced exogenous antigens at high levels without causing overt disease^15^. In addition, humoral immune responses were observed following mucosal exposure of RCN-vectored rabies vaccine in another bat species, *Eptesicus fuscus*^16^.

As a starting point in developing vaccines for WNS, we targeted two potentially immunogenic antigens from *Pd*. The first is calnexin (CAL), a highly conserved ascomycete fungal antigen that has been shown to protect mice against a variety of fungal pathogens^17^. Indeed, mouse T-cells specific for CAL^18^ proliferate in the presence of *Pd*^17^. The other, a subtilisin-like or serine protease (SP), also known as “destructin-1”, was identified in two separate studies as a major proteolytic component of *Pd*^19,20^, likely facilitating tissue invasion of the host. As a major component of the secretome of *Pd*, SP could be a desired target of the host’s specific immune response, as disrupting the activity of the protease may limit the ability of *Pd* to cause disease. Studies have shown that WNS induces anti-fungal immune responses in the skin and lymph nodes of hibernating bats, including an IL-17A response^21,22^. Because IL-17A is known to recruit and activate phagocytes targeting fungi^18^, results suggest that bats are mounting an adaptive immune response to *Pd* during hibernation^21,22^, although there is no evidence that this immunity is protective.

In this study, we assessed whether induction of specific immunity through vaccination can reduce the disease burden of WNS in a highly susceptible host, *M. lucifugus*. We predicted that the development of cell-mediated immune responses to specific antigens prior to infection with *Pd* would limit cutaneous invasion of the fungus, leading to milder disease presentation and greater survival rates among infected individuals. To test this, we conducted two vaccine trials in which we immunized captive *M. lucifugus* bats with different vaccine formulations via different routes of administration and challenged them with *Pd* prior to hibernation.

## Results

### Characterization of RCN expressing *Pd* antigens by PCR and western blot

We first identified a potential CAL-like protein expressed by *Pd* by using the basic local alignment search tool (BLAST, blast.ncbi.nlm.nih.gov) to search the *Pd* proteome for proteins with significant homology to the CAL from *Paracoccidiodes brasiliensis* (*Pb*), previously shown to be immunogenic and protective^17^. We found a 571 amino acid protein that shared 79% sequence similarity and was categorized in the calreticulin superfamily (Genbank: GMDG_03017). We generated RCN constructs expressing *Pd* CAL (RCN-CAL) and confirmed insertion by PCR (Supplementary Fig. S1). Expression was tested *in vitro* by western blot analysis using serum from CAL vaccinated mice (Supplementary Fig. S2). All RCN-CAL constructs produced an antigen specific band that was not present in cells infected with RCN expressing green fluorescent protein (GFP) and corresponded to the major immunoreactive band of 63 kDa from positive control CAL produced in *E*. *coli* previously^17^. Protein was found in the pellet fraction, as expected of a membrane bound protein. We also generated RCN expressing the “destructin-1” serine protease (RCN-SP), as this protein was hypothesized to be involved in development of WNS^19,20^. PCR confirmed insertion of the gene (Supplementary Fig. S1) and qPCR for SP specific RCN transcribed mRNA confirmed expression.

### Vaccination and challenge of bats

#### Pilot vaccine trial

Bats were randomly assigned to groups of 10 or 11 for the following vaccine treatments in mid-November, 2015 (Table 1): CAL protein via intramuscular (IM) injection, RCN-CAL via intranasal (IN) injection, RCN-CAL and RCN-SP simultaneously injected IM, inactivated *Pd* culture injected intraperitoneally (IP), and a control group that received phosphate buffered saline (PBS) via IM injection. Bats were boosted (same route and dose) 22 days post-initial vaccination. Unfortunately, by this time, weight loss, non-specific morbidity, and mortality associated with husbandry issues began to occur (see Supplementary material for more details), complicating the trial. None of the bats developed pox lesions or had any signs of morbidity related to vaccination with RCN or other treatments, but challenge with *Pd* and hibernation were delayed until early February (60 days post-boost) when body condition and weights stabilized. By the time bats were challenged, average body weights of each group ranged from 7.95–9.62 g (but were not statistically different) and numbers of bats in each treatment group were diminished, particularly the control group (Table 1).

**Table 1:** Mean weight (in grams) of *Myotis lucifugus* that received various treatments prior to challenge with *Pseudogymnoascus destructans (Pd)* and post-challenge survival.

Treatment	Route	n	% survival after challenge	Mean weight at challenge (g) ± s.d.
PBS	IM	3	0	8.68 ± 0.21
CAL	IP	7	0	7.95 ± 1.22
RCN-CAL	IN	8	25	9.09 ± 1.12
RCN-CAL/RCN-SP	IM	10	10	8.23 ± 1.28
Inactivated *Pd*	IM	9	44	9.62 ± 1.38

Treatments included phosphate buffered saline (PBS) as a negative control, calnexin (CAL) protein, raccoon poxvirus (RCN) expressing CAL, RCN expressing CAL and RCN expressing serine protease (SP) in combination, and inactvated *Pd*, administered via intramuscular (IM), intraperitoneal (IP), or intranasal (IN) routes.

**Figure 1:**
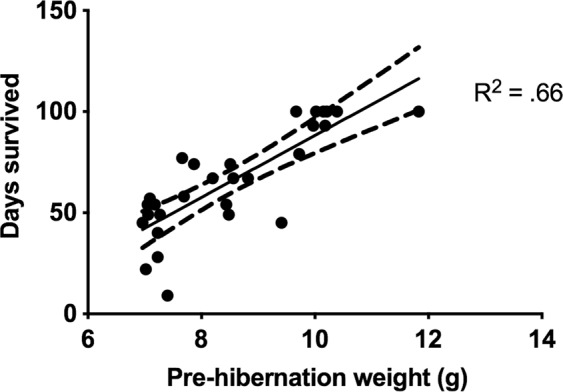
*Myotis lucifugus* survival was correlated to pre-hibernation weight. The number of days bats survived after challenge with *Pseudogymnoascus destructans* is plotted against pre-hibernation weight, with Pearson correlation test line and confidence intervals.

Of 37 bats (31 adults and 6 juveniles) that were challenged with *Pd* and placed into the hibernation chamber, 3 died before day 40 post-inoculation (days 9, 22, and 28) and were excluded from further analyses (see Supplementary Table S1 for details). Only seven bats survived the entire 100-day hibernation period (ending in early May): four that received inactivated *Pd* (one individual was WNS positive), two that received RCN-CAL IN (both WNS positive) and one that received RCN-CAL/RCN-SP (WNS negative). No significant differences in survival were evident between treatment groups, but the numbers of animals in each treatment group were very low. Instead, survival strongly correlated with their weight when placed into the environmental chamber, with bats of lower weight being more likely to die before 100 days regardless of their treatment group (Fig. 1).

**Figure 2:**
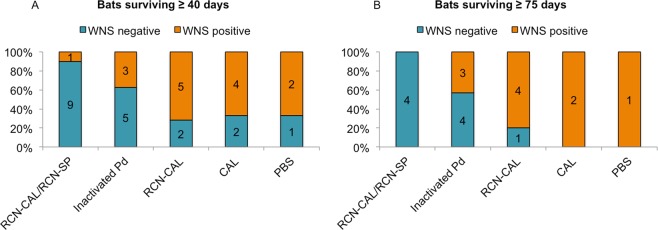
Proportion of *Myotis lucifugus* positive and negative for white-nose syndrome (WNS) by histological examination after challenge with *Pseudogymnoascus destructans (Pd*) in hibernating bats surviving 40 days or more (**A**) or 75 days or more (**B**). Bats received either a combination of raccoon poxvirus (RCN) expressing calnexin (CAL) and RCN expressing serine protease (RCN-CAL/RCN-SP), inactivated *Pd*, RCN-CAL only, purified CAL protein, or phosphate buffered saline (PBS). Numbers indicate the total number of bats in each group.

Fifteen of 34 bats examined developed histologic lesions characteristic of WNS^23^ (Fig. 2) as described in Supplementary materials and depicted in Supplementary Fig. S3). In addition to or in place of characteristic WNS lesions, a few bats had microscopic evidence of a resolving fungal infection, including the formation of neutrophilic pustules containing rare fungal hyphae (Supplementary Fig. S3). Lesions of WNS were evident in one bat found dead as early as day 45 post-challenge. Of those bats that survived past day 40 post-challenge (Fig. 2A), the proportion with WNS lesions was significantly lower (P < 0.05) in the RCN-CAL/RCN-SP group (1/10) compared to the groups that received RCN-CAL (5/7) or CAL protein (4/6), but not the inactivated *Pd* group (3/8). We did not have the power to detect differences between the PBS control group (2/3) and other treatment groups due to the small sample size of this group. For those surviving past 75 days (Fig. 2B), a similar result was obtained using Fisher’s exact test for small samples sizes. The proportion of bats positive for WNS was significantly lower (P = 0.007) in those that received RCN-CAL/RCN-SP (0/4) compared to the proportion of bats (7/8) that received RCN-CAL, CAL, or PBS, but not different than bats vaccinated with inactivated *Pd* (3/7). *Pd* DNA was detected by real-time PCR on all but one of 29 bats swabbed at death or euthanasia (Supplementary Table S1).

#### Second vaccine trial

A second trial was conducted to further evaluate the most promising treatment in the pilot study, RCN-CAL/RCN-SP, for both its protective efficacy after *Pd* challenge and stimulation of anti-fungal immunity. In the second trial, all bats were juveniles, vaccinated the day after intake in mid-October, only once either IM or orally, and maintained in an active state for only 22 days before they were challenged with *Pd* and placed in an environmental chamber for hibernation in November. Controls received RCN expressing luciferase (*luc*), a neutral antigen, to control for the effects of the viral vector (Table 2).

**Table 2:** Percent survival and mean weight (in grams) at challenge and at death or euthanasia in *Myotis lucifugus* vaccinated with raccoon poxvirus (RCN) expressing calnexin (RCN-CAL) and RCN expressing serine protease (RCN-SP) in combination, either via the oral route or intramuscular (IM) route, or RCN expressing luciferase (RCN-*luc*, control group).

Treatment	Route	n	% survival after challenge	Mean weight at challenge (g) ± s.d.	Mean weight at death (g) ± s.d.
RCN-*luc*-control	IM/oral	9	30	10.1 ± 0.9^A^	5.3 ± 1.8^A^
RCN-CAL/RCN-SP	IM	10	80	10.4 ± 1.1^A^	6.1 ± 0.9^AB^
RCN-CAL/RCN-SP	oral	8	88	10.5 ± 1.3^A^	7.0 ± 0.8^BC^

Bats were challenged with *Pseudogymnoascus destructans* three weeks after immunization and just prior to a 120 day hibernation period. Mean weights with different letters within a column are significantly different at P < 0.05.

**Figure 3:**
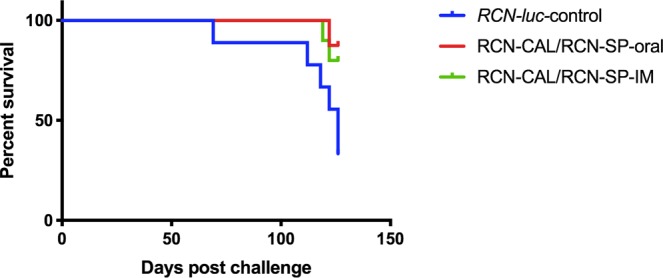
Comparison of survival of vaccinated and unvaccinated *Myotis lucifugus*. KaplanMeier survival analysis shows that bats vaccinated with a combination of raccoon poxvirus (RCN) expressing calnexin (RCN-CAL) and RCN expressing serine protease (RCN-SP), either via the oral route or intramuscular (IM) route, survived at a significantly (P = 0.01) higher rate than control bats receiving RCN expressing luciferase (RCN-*luc*).

Pre-challenge survival of bats in this trial was much better than the previous trial. Only two bats were excluded due to a failure to adapt to captivity (weight < 7 g). Mean weight at the beginning of hibernation was >10 g and not significantly different between groups (Table 2). Overall, post-challenge survival was much higher (18/27) than the previous trial, with vaccinees (regardless of route of immunization) surviving at a higher rate than controls (Fig. 3; P < 0.05). Mean end weight of bats (both live and dead, excluding one control too mummified for evaluation) was significantly higher in the oral vaccinees compared to controls, but IM vaccinees did not differ significantly from either of the other groups (Table 2).

**Figure 4:**
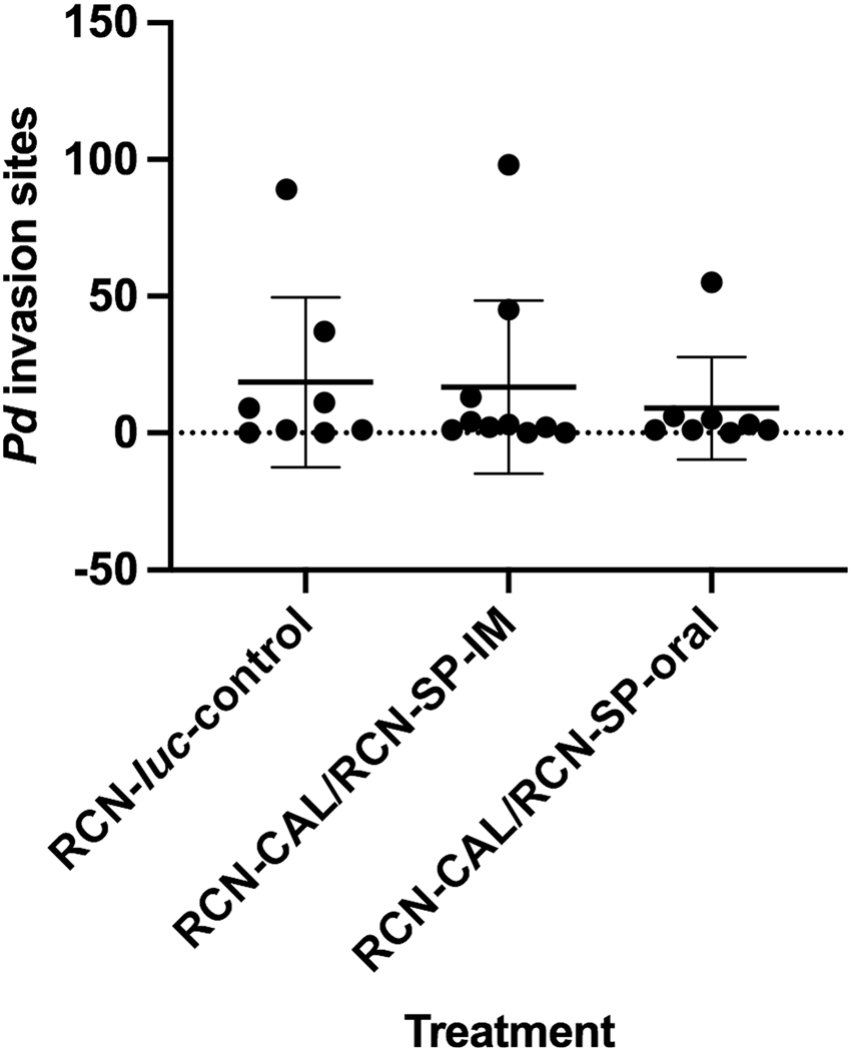
Number of *Pseudogymnoascus destructans (Pd)* invastion sites evident by histology on the wings of vaccinated and unvaccinated *Myotis lucifugus* after *Pd* challenge and hibernation.

All dead bats had evidence of *Pd* infection - lesions of WNS on histologic examination or obvious orange fluorescence under UV light indicative of *Pd* invasion – with the exception of one control bat that was found mummified on day 126 and was too decomposed for evaluation (Supplementary Table S2). All dead bats had lost >47% of their initial body weight (54.7% ± 7.6) and died between day 69 and 122. Most bats surviving to day 126 also had evidence of *Pd* infection (15/18), but they lost significantly (P < 0.0001) less weight (33.9% ± 8.5) compared to those that died. The average numbers of WNS lesions (after removing 3 statistical outliers, one in each treatment group, with >45 invasion sites) in all bats vaccinated with RCN-CAL/RCN-SP via oral (2.4 ± 2.3) or IM (3.1 ± 4.2) routes were lower (Fig. 4) but not statistically different than control bats sham-vaccinated with RCN-*luc* (8.4 ± 13.4).

### *Ex vivo* cytokine expression by bat T cells

To look for evidence of T cell priming and function in surviving bats, we stimulated lymphoid tissue derived cells (pooled spleen and axillary lymph nodes) *ex vivo* and analyzed the cells for intracellular cytokine expression by flow cytometry. Since there are no commercially available anti-bat (i.e. *M*. *lucifugus*) antibodies for CD4, CD8, IFN-γ and IL-17A, we employed Prime Flow technology, which uses fluorescent *in situ* hybridization (Flow-FISH) to quantify gene expression at the mRNA level by flow cytometry. We also used anti-human anti-CD3 antibody that cross-reacts with bat T cells and anti-bat IgG for the detection of B cells^24^. Combined detection of protein (using cross-reactive antibodies) and mRNA (using Flow-FISH) at a single cell level has been described^24^.

**Figure 5:**
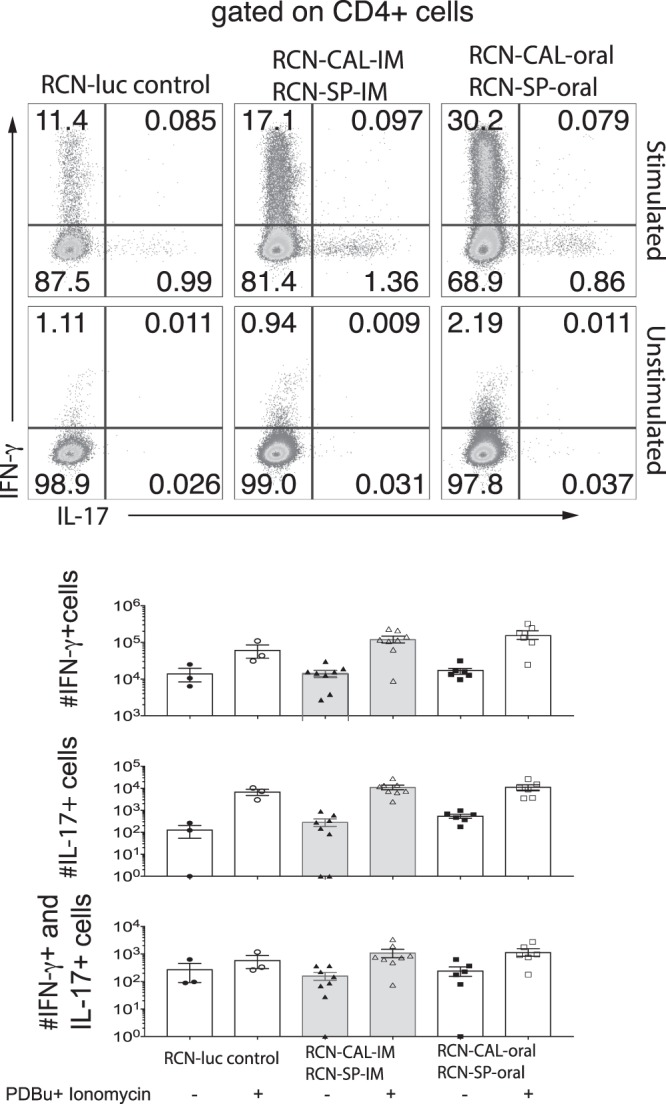
Cytokine expression by bat CD4^+^ T cells measured by Flow-FISH in vaccinated *Myotis lucifugus* that survived challenge with *Pseudogymnoascus destructans (Pd)*. Bats had been vaccinated with raccoon poxviruses (RCN) expressing calnexin and serine protease (RCN-CAL/RCN-SP) via intramuscular (IM; n = 8) or oral (n = 7) administration, or sham vaccinated with RCN expressing luciferase (RCN-*luc*; n = 3). Bat splenocytes were stimulated with PDBu and ionomycin for 2 hours for RNA detection by Flow-FISH. Dot plots and histograms represent concatenates and averages of three to eight bats per group.

Thirty percent or more of the stimulated CD4^+^ and CD8^+^ T cells from bats orally vaccinated with RCN-CAL/RCN-SP produced IFN-γ, whereas less than 1% produced IL-17A (Fig. 5 and Supplementary Fig. S4). The number of stimulated CD4^+^ T cells from orally vaccinated bats that produced IFN-γ was increased (P = 0.07) compared to RCN-*luc* controls (Fig. 5). Similar data were found for bat CD8^+^ cells (Supplementary Fig. 4), but the ability to detect differences between vaccinees and controls was limited by the low number of control bats. These data indicate that vaccination of bats with RCN-CAL/RCN-SP primarily drove a Th1 immune response that was augmented in oral vaccinees compared to controls, but not IM vacinees.

**Figure 6:**
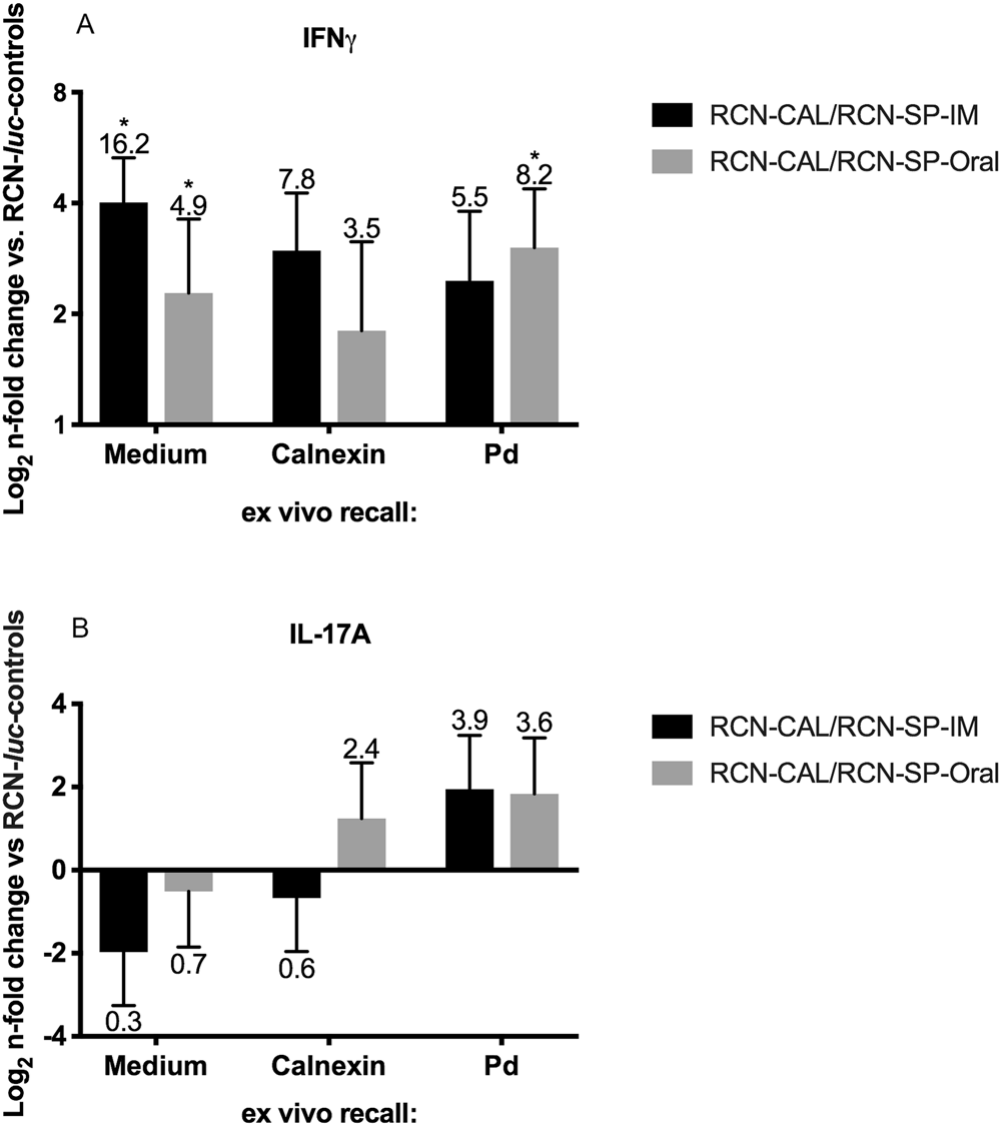
Expression of IFN-γ (**A**) and IL-17A (**B**), measured by RT-PCR, in pooled spleen and axillary lymph nodes of vaccinated *Myotis lucifugus* after challenge with *Pseudogymnoascus destructans (Pd)*, and *ex vivo* recall with calnexin (CAL), *Pd* or medium (non-stimulated). Bats had been vaccinated with raccoon poxviruses (RCN) expressing CAL and serine protease (RCN-CAL/RCN-SP) via intramuscular (IM; n = 8) or oral (n = 7) administration, or sham vaccinated with RCN expressing luciferase (RCN-*luc*; n = 3). Bars and error bars represent average n-fold changes (indicated by the number above the error bar) and standard errors normalized to the RCN-*luc* group. Values marked with an asterisk are significantly different from the RCN-*luc* control group at a P < 0.05.

**Figure 7:**
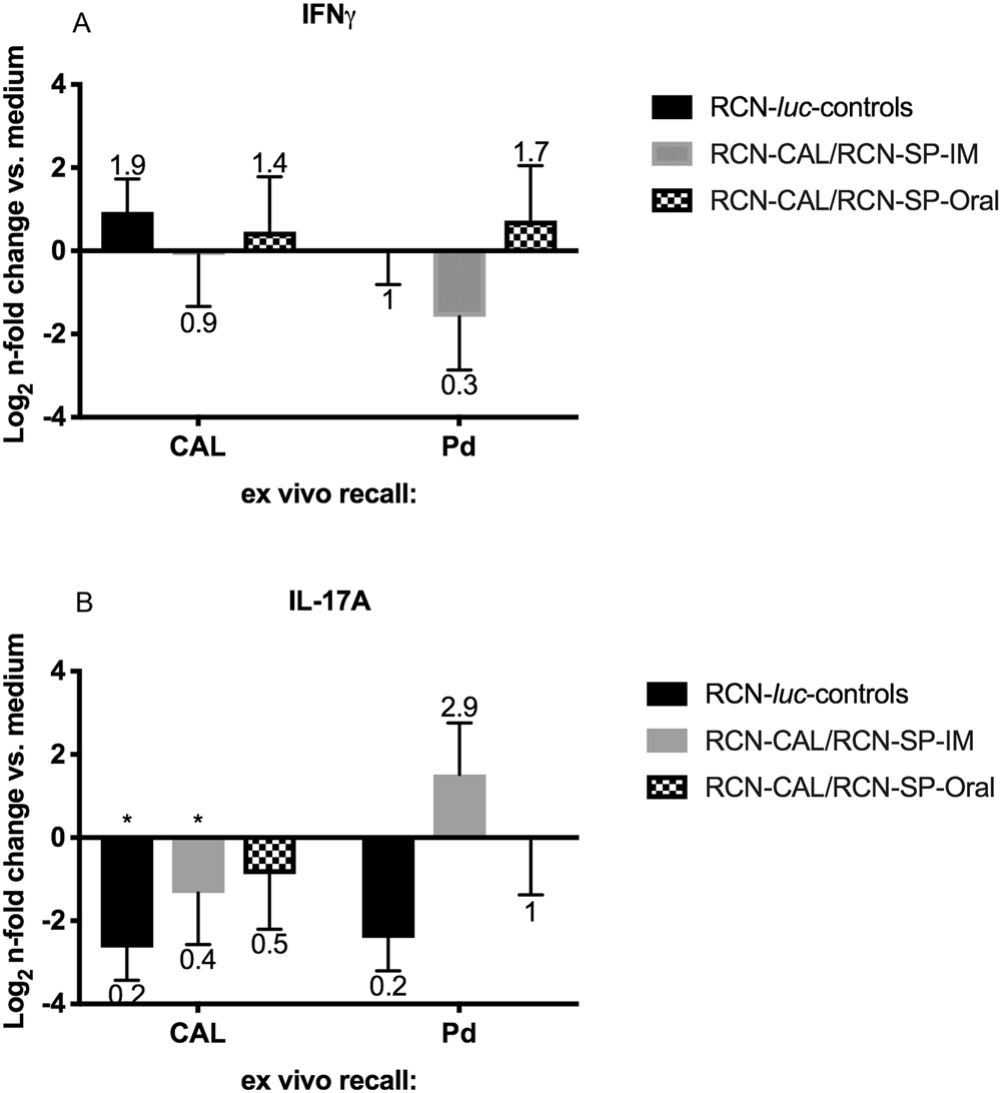
Expression of IFN-γ (**A**) and IL-17A (**B**), measured by RT-PCR, in pooled spleen and axillary lymph nodes of vaccinated *Myotis lucifugus* after challenge with *Pseudogymnoascus destructans (Pd)* and *ex vivo* recall with calnexin or *Pd*. Bats had been vaccinated with raccoon poxviruses (RCN) expressing calnexin and serine protease (RCN-CAL/RCN-SP), via intramuscular (IM; n = 8) or oral (n = 7) administration, or sham vaccinated with RCN expressing luciferase (RCN-*luc*; n = 3). Bars and error bars represent average n-fold changes (indicated by the number above the error bar) and standard errors, normalized to the non-stimulated (medium) control. Values marked with an asterisk are significantly different from the medium control at P < 0.05.

Since we used PDBu and ionomycin to stimulate bat T cells *ex vivo*, cytokine expression measured by Flow-FISH is due to all T cells that have been primed in the bats. To measure vaccine antigen-specific T cell responses, we restimulated primed lymphoid tissue derived cells *ex vivo* with CAL or inactived *Pd* for 48 h and measured cytokine transcript by RT-PCR. We analyzed and illustrated cytokine transcript expression relative to two denominators: 1) the RCN-*luc* control group and 2) medium control stimulation. Figure 6 shows the n-fold changes in cytokine expression vs. the RCN-*luc* control group. IFN-γ transcripts were elevated (P < 0.05) in bats vaccinated orally and IM with RCN-CAL/RCN-SP compared to those that received RCN-*luc*, independent of the *ex vivo* stimuli (medium, CAL and inactivated *Pd*), indicating that T cells had been induced *in vivo* to express the cytokine in a vaccine antigen-specific manner. The expression of IL-17A was not significantly altered in vaccinated vs. control bats. Figure 7 shows the n-fold changes in cytokine transcripts after *ex vivo* stimulation with CAL and inactivated *Pd* vs. medium control. The robust expression of IFN-γ in the RCN-CAL/RCN-SP vaccine recipients (Figs 6A and 7A) could not be further increased by *ex vivo* stimulation with CAL and inactivated *Pd*. In summary, the transcript analysis by Flow-FISH and real-time RT-PCR indicate a strong induction of IFN-γ expression by administration of RCN-CAL/RCN-SP.

## Discussion

Currently, few mitigation strategies are in use for limiting the spread and lethality of WNS in bats in North America. The trials described here are the first steps toward the development and testing of vaccination as a potential control method for WNS. Despite challenges in maintaining *M*. *lucifugus* in captivity, several findings are of note. Most importantly, rates of survival were higher in bats vaccinated with RCN-CAL/RCN-SP compared to controls that were challenged with *Pd* and placed in a hibernation chamber for 126 days. Orally vaccinated bats, in particular, had higher mean weights at death or euthanasia compared to controls, though all had evidence of *Pd* infection, and they had higher numbers of CD4 + T-cells producing IFN-γ. Similarly, in our pilot trial, a higher proportion of bats vaccinated with RCN-CAL/RCN-SP remained WNS negative 75 or more days post-challenge compared to other treatment groups, although the number of controls was small in that study. Further work is needed, but the combined results from the two trials provide evidence that vaccination can potentially protect bats from the effects of *Pd* infection. Vaccination may have slowed the growth of the fungus or reduced the severity, persistence, or discomfort of WNS lesions, thus decreasing arousal of bats during hibernation and enhancing their survival, even if the prevalence of the fungus on bats was unaffected.

Recent studies^22,25^ suggest that antibody-mediated immunity alone may not be protective against WNS, but until now, other mechanisms of immunity had not been studied. For several pathogenic mycoses, cell mediated immune responses have been shown to be crucial for protective immunity^18,26,27^. We employed Flow-FISH for the first time on *M*. *lucifigus* tissue to quantify immunologic responses on a per cell basis, because no commercial antibodies are currently available to demark little brown bat-specific CD4^+^ or CD8^+^ T cells and IFN-γ or IL-17A. Although we observed a trend (P = 0.07) towards increased expression of IFN-γ in surviving bats orally vaccinated with RCN-CAL/RCN-SP, the power to detect statistical differences between the vaccinees and controls was limited by the low numbers of surviving controls. Antigenic *ex vivo* stimulation with CAL and inactivated *Pd* indicated elevated IFN-γ transcripts in bats vaccinated orally and IM with RCN-CAL/RCN-SP compared to controls that received RCN-*luc*, independent of the *ex vivo* stimulus (medium, CAL and inactivated *Pd*), indicating that the T cells had been induced *in vivo* to express the cytokine in a vaccine antigen-specific manner. These cellular immune responses alone, or combined with antibody responses, may offer a basis by which recombinant RCN-CAL/RCN-SP may protect against WNS.

It is possible that some of the bats included in our initial trial may not have been naïve to *Pd* as the majority were adults (31/37 challenged) and evidence of *Pd* infection in the trapping location of our study bats was found the previous spring. If adult bats were previously primed to *Pd*, vaccination could have boosted their immune response. Alternatively, pre-existing exposure could potentially result in a maladaptive response to *Pd* with subsequent tissue damage and increased numbers of WNS cases. This latter outcome seems unlikely, as similar responses would have been expected in all three groups that received CAL, and that was not the case. Of the vaccinated adults, 80% of bats that received CAL (n = 5) and 67% that received RCN-CAL (n = 6) developed WNS, compared to 11% of bats that received RCN-CAL/RCN-SP (n = 9). In our second vaccine trial, only juveniles were included, as young animals typically respond more vigorously than adults to vaccination, including with other RCN vaccines^28^. Also, juveniles would not have been exposed to *Pd* the prior season, eliminating that variable.

Recombinant viral-vectored vaccines, such as RCN, have been shown to stimulate broad humoral and cellular immunity that provide long lasting protection^15,29–33^, particularly via mucosal delivery. Thus it is not surprising that oral vaccination appeared to be somewhat more effective than IM vaccination in our study. RCN has been shown to be safe and effective in a variety of species via the oronasal route, including domestic cats, piglets, dogs, raccoons, skunks, foxes, bobcats, rabbits, sheep, prairie dogs, non-human primates, chickens and bats, with none of the immunized animals showing clinical side effects^15,30,34–42^. Recently, a bait-delivered RCN-based sylvatic plague vaccine was evaluated in field trials in prairie dog colonies, demonstrating higher relative abundance and survival of prairie dogs on vaccine treated plots compared to paired placebo plots^14^. More importantly, an RCN-rabies vaccine construct was shown to protect *E*. *fuscus* (big brown bats) from rabies challenge after vaccination via both oronasal and topical application^16^, providing further support that an orally delivered RCN-based WNS vaccine may be feasible, once the most protective antigens are identified. In addition to CAL and SP, we are testing additional *Pd* antigens that could help slow the growth of *Pd* or reduce symptoms of infection that increase arousal of bats during hibernation, reducing their critical energy stores. Bats are naturally fastidious and spend a large amount of time grooming themselves^43^. Because of this, efficient vaccination of large groups of bats may be accomplished by applying an oral WNS vaccine topically in a liquid or paste vehicle, which would be consumed by bats during grooming.

In addition to identifying protective *Pd* antigens, other factors must be considered for further development of an RCN-based vaccine against WNS. Topical application will require an effective delivery vehicle that maintains vaccine titers, attaches well to the fur of target bat species, is sufficiently palatable to induce ingestion of the vehicle-vaccine mixture, and contains a tractable biomarker that allows easy distinction between vaccinated/unvaccinated animals. In addition, the optimal dose, number of applications, and timing of vaccination would need to be determined. These factors would be best determined in controlled field trials as maintaining insectivorous bats in captivity for long periods of time has proven difficult.

During our initial trial, problems were encountered in keeping *M*. *lucifugus* alive and healthy in a bio-secure environment, leading to diminished group sizes (see Supplement). The nature of the clinical signs and the fact that the greatest losses were encountered in the negative control group that received an IM dose of PBS, suggested that husbandry issues, particularly improper environmental humidity, were primarily responsible for the observed health problems, decreased feeding behavior, and weight loss, rather than the vaccines administrated. No evidence of poxvirus disease was seen clinically or on histologic evaluation in either study, and RCN has been shown to be safe in other bat species^15^. While most problems resolved prior to challenge in the pilot study, many of the bats apparently did not regain the levels of fat reserves necessary for hibernation, which likely explains the correlation between starting body weight and survival time during the challenge (Fig. 1). Reducing the time bats were kept in an active state in captivity to 3 weeks after vaccination in our second trial, significantly improved survival rates both pre- and post-challenge. This provides further confirmation of the safety of RCN for bats, and also suggests vaccination in the fall just prior to hibernation is a viable option for vaccinating bats in the field.

Measures for controlling WNS are crucially needed to help mitigate the loss of bat species threatened by this disease. Here we present several potential WNS vaccine candidates and preliminary evaluation of their safety in bats and efficacy in preventing WNS. Most importantly, these studies provide some evidence that immunity to WNS is possible, and therefore further development and testing of potential vaccines against *Pd* is warranted.

## Materials and Methods

### Ethical statement

Bats were captured and brought into captivity under a permit obtained from the Wisconsin Department of Natural Resources (WDNR), Endangered and Threatened Species (E/T) permit #922. All experimental procedures on bats were reviewed, approved, and performed in accordance with all relevant guidelines and regulations under University of Wisconsin (UW) animal care and use committee (protocol #V005277) or the US Geological Survey, National Wildlife Health Center (NWHC) animal care and use committee (protocol # EP170719). Construction of recombinant vaccine viruses was reviewed and approved by UW biosafety committee (protocol #B00000236).

### Construction of RCN based vaccines

RCN was cultured in African Green Monkey kidney cells (ATCC #CCL-81) grown in Dulbecco’s modified Eagle medium (DMEM) supplemented with 10% fetal bovine serum, 2 mM L-glutamine, 1.5 g/l sodium bicarbonate, 100 U/ml of penicillin, 100 μg/ml of streptomycin, and incubated at 37 °C in 5% CO_2_. Wild-type RCN was provided by the Centers for Disease Control (Atlanta, GA); the RCN-luciferase (*luc*) strain was previously described^40^.

Recombinant RCN viruses expressing *Pd* CAL (Genbank: GMDG_03017, RCN-CAL) or serine protease (Genbank: GMDG_06417, RCN-SP) were constructed as described previously^31^. Protein sequences were back-translated and codon-optimized for expression in vaccinia virus, then commercially synthesized. Genes were cloned into RCN specific transfer vectors to express in frame with a tPA secretory signal coding region and under the control of a poxvirus synthetic early-late promoter, with an internal ribosomal entry site expression enhancing element as previously described^31^. Expression was evaluated by western blot assay or RT-PCR.

### Preparation of inactivated *Pd* and subunit protein vaccine

Generation and purification of CAL from *Paracoccidiodes brasiliensis* (*Pb*) was described previously^17^. Calnexin from *Pd* was cloned into the pET-28 expression by replacing the coding region of the *Pb* CAL with that of the *Pd* CAL by overlap extension PCR^44^. To generate inactivated *Pd*, cultures were grown as described below and hyphae and conidia harvested by scraping fungal material with a metal pick. The fungus was pulverized into a very fine powder in liquid nitrogen using a mortar and pestle and weighed. Petri dishes containing *Pd* were placed on a 212 nm ultraviolet light imager for 30 minutes to kill the fungus. The pulverized material was then suspended in cold PBS to a concentration of 400 mg/ml, and drawn through successively finer syringes until liquid was able to be easily drawn through a 27 gauge needle.

### Preparation of *Pd* inoculum

Sabouraud dextrose plates containing chloramphenicol and gentamycin were inoculated with a mycelial plug from a starter culture of *Pd* and incubated for approximately 60 days at 7–10 °C. Sterile PBS containing 0.5% Tween-20 (PBST) was used to wash colony surfaces, followed by gentle rubbing with a sterile inoculating loop to dislodge conidia. Liquid containing conidia was transferred to a sterile tube on ice and then centrifuged at 3,500 RCF for 8 min; the supernatant was removed and the pellet re-suspended in cold sterile PBST. The suspension was passed through a 20 gauge needle several times to break apart aggregations. Conidia were enumerated using a hemocytometer, and the concentration adjusted to 2.5 × 10^4^ conidia per μl using sterile PBST.

### Vaccination and challenge of bats

#### Experimental animals

The first vaccine trial was conducted at the UW School of Veterinary Medicine, Charmany Instructional Facility (CIF). The second trial was conducted at NWHC.

For the first and second vaccine trials, male *M*. *lucifugus* were wild-caught at hibernacula in Dodge or Pierce County, Wisconsin, respectively by representatives of WDNR in late fall. Both adults (n = 44) and juveniles (n = 8) were captured for the first trial, but only juveniles (n = 29) were captured for the second trial. Wing bands were placed on each bat for identification. The bats were maintained under ABSL-2 conditions in 40 × 76 × 122 cm (378 liter) screen flight cages, kept within a 1.7 × 2.0 m greenhouse tent to allow increased humidity (85–95%) using warm mist humidifiers. Temperatures were maintained at 21–24 °C. On intake, bats were treated topically for parasites with selamectin. Bats were fed mealworms (*Tenebrio molitor*) supplemented with a vitamin and mineral mixture (Vionate®); water was available *ad libitum*. They were weighed frequently and any bats that had lost >0.3 g of body weight were hand fed with mealworms or liquid Carnivore Care™ diet if mealworms were refused.

#### Pilot vaccine trial

Bats were randomly assigned to groups of 10 or 11 for the following vaccine treatments in mid-November, 2015 (Table 1). One group (n = 10) received RCN-CAL at a dose of 10^8^ plaque-forming units (PFU) administered IN under light anesthesia with isoflurane by drop-wise pipetting into both nostrils. A second group (n = 11) received both RCN-CAL and RCN-SP (10^8^ PFU of each) injected IM into each separate rear deltoid muscle (RCN-CAL/RCN-SP). A third group (n = 10) was injected IM with an inactivated *Pd* preparation (20 mg in a volume of 50 µl). A fourth group (n = 11) received recombinant CAL protein (40 µg of both *Pb* and *Pd* CAL), mixed with alum in a total volume of 0.2 ml and injected IP. A negative control group (n = 10) was injected IM with PBS. Bats were boosted (same route and dose) 22 days post-initial vaccination. During vaccination, treatment groups were housed in separate cages to avoid cross infection with RCN constructs.

Due to health issues with the bats and difficulties in maintaining weight (see Supplement for details), the challenge was delayed until early February 2016. Bats (n = 37) were challenged with *Pd* 60 days after boosting. *Pd* inoculum was freshly prepared as above and kept on ice and in the dark. Before application to each bat, suspensions were thoroughly mixed by pipetting. When the mixture was uniform, 20 µL were pipetted onto the dorsal surface of the right wing between the 5^th^ digit and the forearm while gently using the pipet tip to spread the liquid evenly across this area, so as not to damage the bat’s wing with the tip of the pipet. Once the liquid had been evenly spread, the wing was allowed to fold back into its resting position. Bats were then placed in an environmental chamber designed to mimic the conditions of stable low temperatures with high humidity found in caves during hibernation (Percival Model #I36NL). The chamber was maintained at 8.5 °C (±0.5 °C) and 90% (±1.0%) relative humidity. Bats were monitored daily by viewing through a window in the chamber door using red light to illuminate the interior. Any bats noted as down on the cage floor for >24 hours were retrieved and a full necropsy was performed. At 100 days, any surviving bats were euthanized and necropsied.

#### Seccond vaccine trial

Twenty-nine bats were randomly assigned to 3 treatment groups and immunized the day after capture. One group received both the RCN-CAL and RCN-SP vaccine constructs (10^7^ PFU of each) via IM injection (n = 10) into each separate rear deltoid muscle (RCN-CAL/RCN-SP-IM). A second group received both constructs (same dosage) via oral administration (n = 9) in a 100 ul volume (RCN-CAL/RCN-SP-ORAL). The third group received RCN-luciferase (*luc*) via IM injection or oral administration, as a negative control, and results were combined for analysis. The bats were housed in separate cages to avoid cross contamination and hand fed every other day as described above. Three weeks after vaccination, bats were challenged with *Pd* as described above and randomly assigned into one of two cages, stratified by treatment, within an environmental chamber. The chamber was maintained at 7.0 °C (±0.5 °C) and 95% (±1.0%) relative humidity. Bats were monitored daily as described above, and at 126 days, surviving bats were euthanized and necropsied.

### Verification of WNS

All bats found dead or euthanized at the end of each trial were examined for evidence of WNS via several methods.

#### Histopathology

We processed the portion of skin connecting the forelimbs with the hindlimbs (plagiopatagia) for histological assessment of WNS status as previously described^23^. Briefly, we dissected the plagiopatagia from both wings, rolled them onto 2 paraffin dowels 2.5 cm in length, fixed them in 10% neutral buffered formalin, trimmed into six sections equal in thickness, dehydrated, embedded in paraffin, sectioned at 5 μm, and stained for light microscopic examination with periodic acid-Schiff (PAS) staining method. This preparation technique yields slides with six whorls of wing membrane from each plagiopatagium. In the pilot trial, a board-certified veterinary pathologist (JL) screened all wing rolls by light microscopy and designated bats as positive or negative for WNS based on published criteria^23,45,46^. In the second study, a board-certified pathologist (MIA) screened by light microscopy a total of six whorls from the right wing of each bat included in the study at 400X for WNS lesions, using the same published criteria. When there were less than 6 rolled sections from the right wing available due to tissue processing artifacts, a complementary number of randomly selected sections were examined from the left wing. The number of WNS lesions in 6 rolls was counted for each bat. Briefly, WNS lesions in both vaccine trials included epidermal invasion by PAS-positive hyphae morphologically compatible with *Pd* forming, distinct cupping erosions (intact epidermal basement membrane) or ulcers (disrupted basement membrane and dermal invasion).

#### Ultraviolet light (UV) transillumination

In the second trial, we examined the wings of each bat using a 366 nm UV lamp and recorded the presence or absence of distinct orange–yellow fluorescence^47^, as well as the visual estimated percentage of wing surface affected (Supplementary Table S2).

#### Quantitative PCR

During necropsy, we swabbed both wings, placed the swabs in sterile water and stored them at −20 °C. Upon thawing, DNA was extracted using 250 μl of PrepMan® Ultra Sample Preparation Reagent and 100 mg of zirconium/silica beads; bead-beating steps were conducted using a FastPrep®-24 homogenizer. The presence of *Pd* DNA was determined using a quantitative real-time TaqMan polymerase chain reaction test targeting the intergenic spacer region of *Pd* as described elsewhere^48^. Samples were considered positive with a threshold cycle (Ct) less than 40.

### Characterization of the adaptive cellular immune response

Following euthanasia of the 18 bats that survived until the end of the second trial, we collected spleen and axillary lymph nodes from each. Splenocytes were collected and pooled with lymph node derived cells (lymphoid tissue derived cells). These pooled cells were used for the following assays:

#### Cytokine expression by bat T cells in peripheral lymphoid tissue using Flow-FISH

Single cell suspensions prepared in complete medium (RPMI + 10% FBS) were incubated with 50 nM PDBu and 0.5 µg/ml ionomycin for 2 hours before cytokine expression was measured by Flow-FISH. Stimulated cells were stained with fixable viability dye and surface antibody including anti-bat IgG (stains B cells). The intracellular detection of T cells with anti-CD3 antibody and staining with the PrimeFlow Target probes for CD4, CD8, IL-17 and IFN-γ was performed using PrimeFlow RNA assay kits and probe sets (Thermo Fisher Scientific) following the manufacturer’s protocol. The samples were recorded by flow cytometry and analyzed by FlowJo software. The gating strategy is shown in Fig. S4A. Briefly, single, live cells were gated for CD3^+^ cells (excluding B cells), then separated into CD4^+^ and CD8^+^ T cells and analyzed for IL-17A and IFN-γ production.

#### Cytokine transcript analysis in peripheral lymphoid tissue using quantitative RT-PCR

We incubated aliquots of 1 × 10^6^ lymphoid tissue derived cells from each surviving bat (n = 18) with 10 µg/ml recombinant *Pd C*AL or with 1 mg/ml heat-inactivated *Pd* extract or 10% FBS/RPMI medium alone (unstimulated control) for 48 h at 37 °C, 5% CO_2_. After incubation we extracted the RNA using Qiagen RNeasy kits. Thereafter, genomic DNA was digested with Turbo DNase. Subsequently we carried out a second round of RNA purification over RNeasy columns before generating cDNA using iScript cDNA kit. Quantitative reverse RT-PCR was performed using SsoFast EvaGreen Supermix with the following primers optimized for little brown bat transcripts: IL-17A forward primer 5′-GCTTCTGTGAGAACTTCCTC-3′; IL-17A reverse primer 5′-CTTGTCCTCAGTATTTGGGC-3′; IFN-γ forward primer 5′-ACAGCAGCAACAGCAAAATG-3′; IFN-γ reverse primer 5′- TTTCCGCATCTTTGGGTTAG-3′. We used the following thermal cycle profile: 30 sec at 95 °C, 45 cycles for polymerase activation, 45 PCR cycles at 95 °C/10 sec (denaturation) and 55 °C/10 sec (annealing/extension), and melt curve (55 °C to 95 °C) on a Qiagen Rotor-Gene Q. Finally, we calculated the n-fold change of gene expression for stimulated (CAL or *Pd* extract) vs. non-stimulated (medium) lymphoid tissue derived cells using the 2^−ddCt^ method^49^ normalizing the Ct values using the highly conserved mouse ortholog 18 S gene with forward primer 5′-CGCCGCTAGAGGTGAAATTCT-3′; reverse primer 5′-CGAACCTCCGACTTTCGTTCT-3′.

### Statistical analyses

For challenge studies, survival among groups was compared using Kaplan-Meier survival curves analyzed by the log-rank test (Graph Pad Prism). Differences in the proportion of bats with WNS lesions evident upon histological examination were analyzed between treatment groups by regression using the package “rms” in R and contrast.rms to run specific contrasts of interest^50^ available from https://CRAN.R-project.org/package=rms or Fisher’s exact test for small sample sizes^51^. Mean number of *Pd* invasion sites identified by histology was analyzed between groups using ANOVA; values identified as outliers were removed. Flow-FISH and real-time RT-PCR data were statistically analyzed in Prism using Welch’s unpaired t-test, which does not assume equal standard deviation; values identified as outliers were removed. Preliminary analyses of survival and proportions of bats with WNS lesions in the second experiment did not detect a significant treatment effect by cage within the chamber, so the data were combined for further analyses. The datasets generated and/or analyzed during the current study are available in the USGS Science Base repository, 10.5066/P923NSSG.

## Supplementary information

Supplementary Methods, Figures, and Tables
