# Supplementary material for: Virally-vectored vaccine candidates against white-nose syndrome induce anti-fungal immune response in little brown bats (Myotis lucifugus)
Source: Sci Rep. 2019 May 1;9:6788. doi: 10.1038/s41598-019-43210-w (PMC6494898; doi:10.1038/s41598-019-43210-w)
Supplement: Supplementary file 1 — Supplementary Methods, Figures, and Tables [file 41598_2019_43210_MOESM1_ESM.pdf]

Virally-vectored vaccine candidates against white-nose syndrome induce anti-fungal immune response in little brown bats (*Myotis lucifugus*)

Supplementary Methods, Figures and Tables

Tonie E. Rocke, Brock Kingstad-Bakke, Marcel Wüthrich, Ben Stading, Rachel C. Abbott, Marcos Isidoro-Ayza, Hannah E. Dobson, Lucas dos Santos Dias, Kevin Galles, Julia S. Lankton, Elizabeth A Falendysz, Jeffrey M. Lorch, J. Scott Fites, Jaime Lopera-Madrid, J. Paul White, Bruce Klein, Jorge E. Osorio

<u>Bat husbandry issues.</u> Maintaining wild-caught *M. lucifugus* in an active state in captivity proved to be very difficult, and mortality unrelated to treatment occurred in our first trial. After vaccination but prior to challenge of bats with *Pd*, signs of weight loss, hair loss, and patchy depigmentation on many of the bats' wings, legs, and face were observed in all treatment groups, including controls. Circumferential areas of constricted tissue appeared on many of the digits, with swelling and redness distally. These lesions were attributed to insufficient humidity. Bats with low body weights received supportive fluids and were hand fed, but despite these efforts, mortalities occurred, primarily in the control animals given IM injections of PBS (7/10 bats died), followed by the group given CAL IP (4/11 bats), RCN-CAL IN (2/10 bats), inactivated *Pd* (1/10 bats), and the RCN-CAL/RCN-SP group (1/11 bats). The PBS control group was housed in a separate cage to avoid potential cross infection with RCN; it was located closest to an evaporative humidifier that was initially used in the greenhouse until we recognized (too late) that it caused dehydration in this group, exacerbating their health issues. This humidifier was replaced with warm mist humidifiers. None of the bats developed pox lesions or had any signs of

morbidity related to vaccination with RCN constructs, and we concluded that husbandry issues were the primary cause of the mortality

Figure S1. A: PCR detection of calnexin DNA fragment inserted in raccoon poxvirus (RCN). B: PCR detection of serine protease DNA fragment inserted in RCN. Viral DNA was extracted from Vero cells infected with either RCN-CAL or RCN-SP by using Quick-DNA viral kit (Zymo Research). OneTaq DNA polymerase (New England Biolabs, Cat# M0486S) and primers pTK-detecting-F and pTK-detecting-R were used to amplify specific DNA fragments containing IRES-tPA-CAL or IRES-tPA-SP. PCR products were subjected to agarose gel electrophoresis and individual images of IRES-tPA-CAL or IRES-tPA-SP gels were captured using UVP Visidoc-it imaging system without manipulations or image processing in compliance with the digital image and integrity policies.

A.

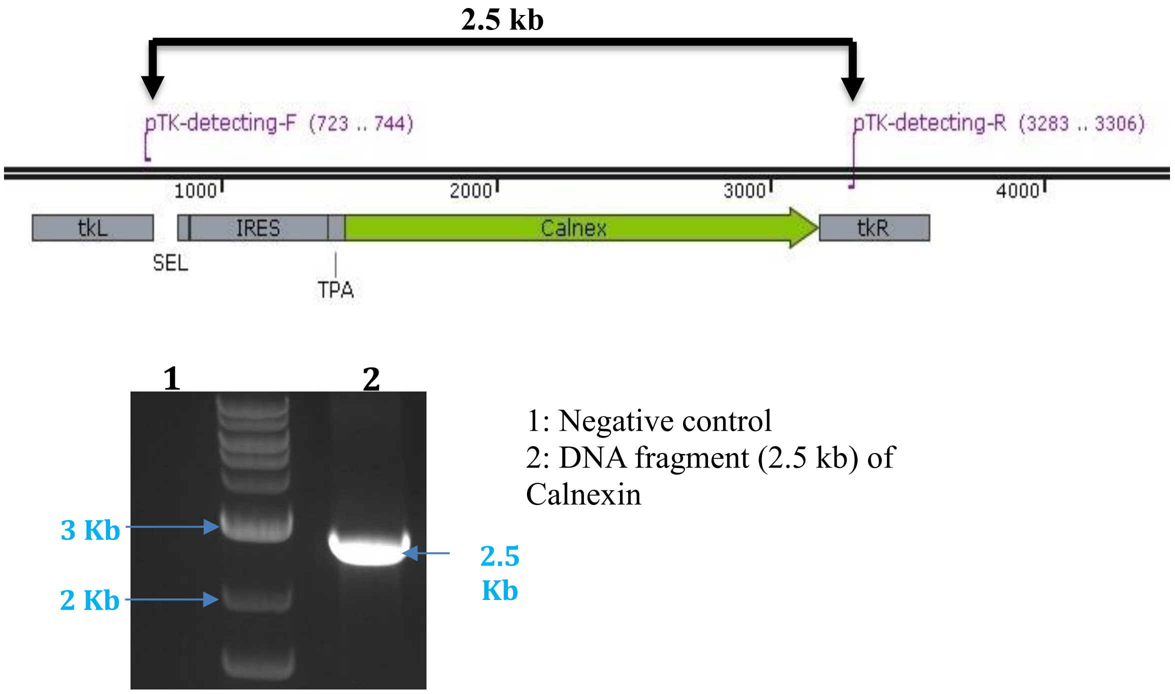

B.

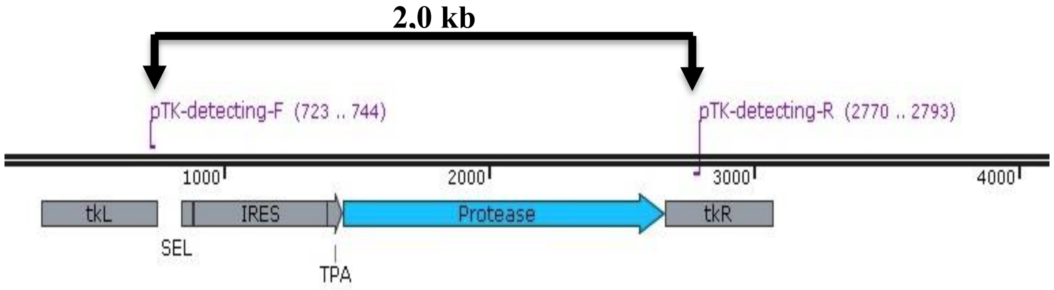

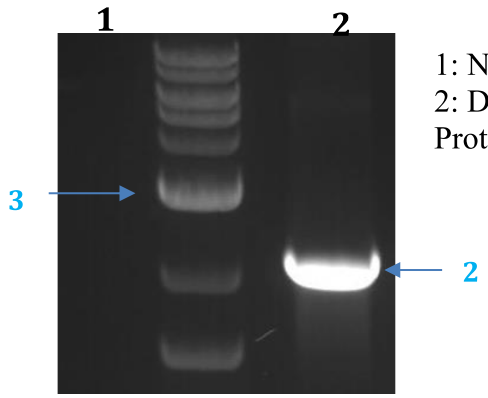

1: Negative control
2: DNA fragment (2.0 kb) of Serine Protease

Figure S2. Western blot for RCN-CAL. Proteins were transferred to a PVDF membrane using Trans-Blot Turbo transfer system (Bio-Rad). Then, the membrane was incubated for 24 hours with serum from CAL vaccinated mice (1:1000 dilution) at 4°C, and then incubated for 2 hours with goat anti-mouse IgG-HRP (Invitrogen, cat# 31430) (1:5000 dilution). Proteins were detected after adding TMB substrate (Novex HRP Chromogenic substrate, Invitrogen) to membrane. Precision Plus Protein Kaleidoscope (Bio-Rad, #1610375) was included to determine protein sizes. Image of the whole membrane was acquired using Epson Perfection 4490 Photo scanner without manipulations or image processing in compliance with the digital image and integrity policies.

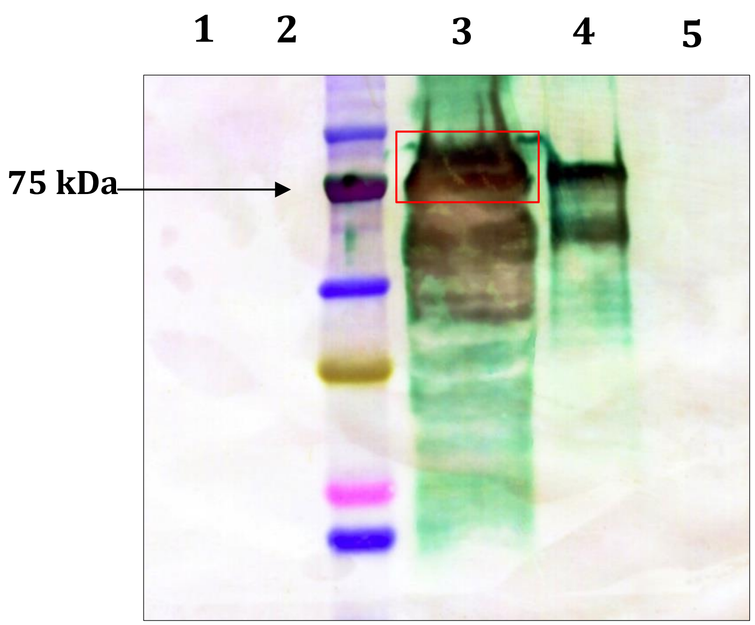

1: RCN control (supernatant)
2: RCN-Calnexin (supernatant)
3: RCN-Calnexin (pellet)
4: Calnexin (purified protein) (Klein lab)
5: RCN control (pellet)

Histology. Bats classified as positive for WNS had characteristic lesions of the disease, including cupping erosions containing periodic-acid Schiff (PAS)-positive fungal hyphae with no inflammatory response (22). In most cases, variable numbers of fungal hyphae extended into or through the dermis (Fig. S3A). Histologic changes in the wing membrane of four of the surviving bats suggested the possibility of resolved or resolving infection. These changes included neutrophilic pustules with rare fungi and hyperpigmentation (Fig. S3B).

Figure S3. A. Lesion of white-nose syndrome (WNS) from bat #1447 euthanized at the end of the study. Fungal hyphae fill epidermal erosions and multifocally extend into the dermis. Periodic acid-Schiff stain, 400X. B. Suspect resolving WNS lesion from bat #1449 euthanized at

the end of the study. The wing membrane contains multiple epidermal pustules with neutrophils and hyperpigmentation. Periodic acid-Schiff stain, 100X. Inset: Pustules contain rare fungal hyphae. Periodic acid-Schiff stain, 400X.

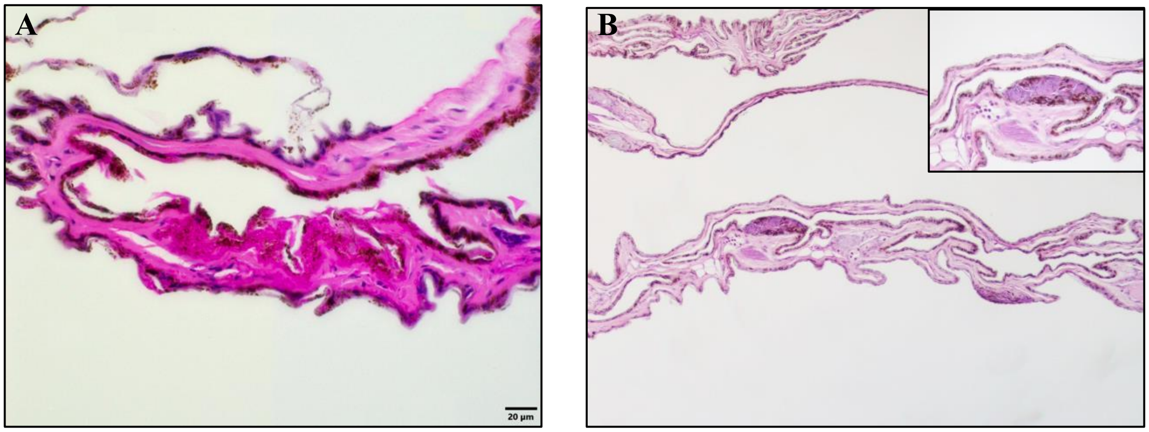

Figure S4A. Gating strategy for Flow-FISH analysis. Live, single cells were gated on CD3$^+$ T cells that were then separated into CD4$^+$ and CD8$^+$ subsets and analyzed for cytokine expression.

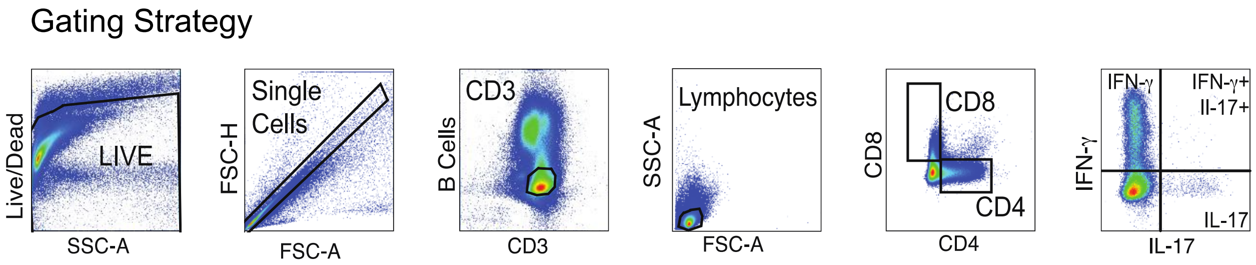

Figure S4B. Cytokine expression by bat CD8$^+$ T cells measured by Flow-FISH. Bat splenocytes were stimulated with PDBu and ionomycin for 2 hours for RNA detection by Flow-FISH. Dot plots and histograms represent concatenates and averages of three to eight bats per group.

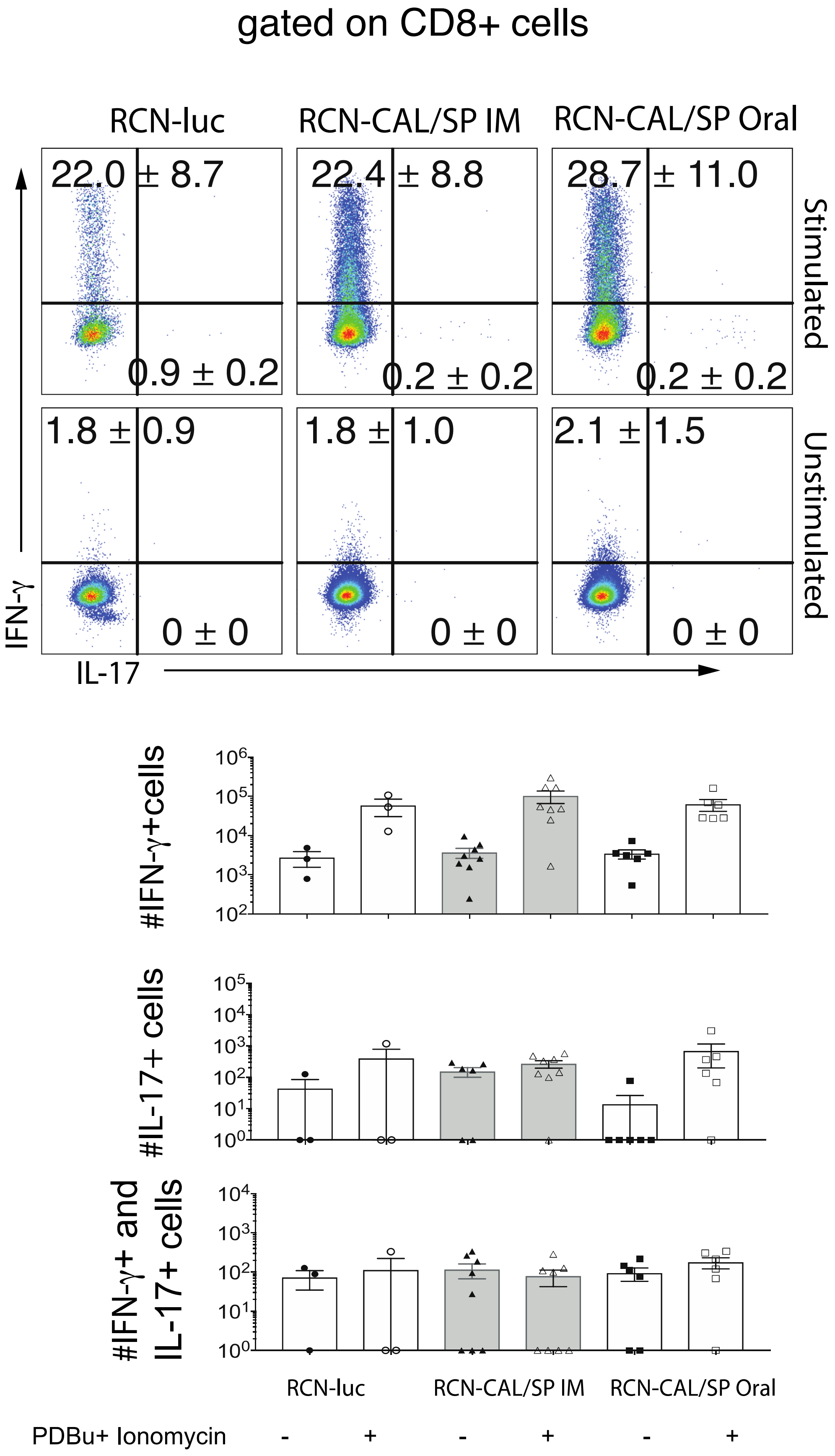

gated on CD8+ cells
RCN-luc
RCN-CAL/SP IM
RCN-CAL/SP Oral
22.0 ± 8.7
0.9 ± 0.2
22.4 ± 8.8
0.2 ± 0.2
28.7 ± 11.0
0.2 ± 0.2
Stimulated
1.8 ± 0.9
0 ± 0
1.8 ± 1.0
0 ± 0
2.1 ± 1.5
0 ± 0
Unstimulated
IFN-γ
IL-17
#IFN-γ+cells
#IL-17+ cells
#IFN-γ+ and IL-17+ cells
RCN-luc
RCN-CAL/SP IM
RCN-CAL/SP Oral
PDBu+ Ionomycin
-
+
-
+
-
+

Table S1. Data collected from *Myotis lucifigus* vaccinated with different treatments and then challenged with *Pseudogymnoascus destructans* (*Pd*) just prior to hibernation. All bats were male (M), either adult (A) or juvenile (J). Treatments included calnexin (CAL), phosphate buffered saline (PBS), inactivated *Pd*, raccoon pox virus (RCN) expressing CAL or serine protease (SP) in combination or RCN-CAL alone. Route of administration was intramuscular (IM), intraperitoneal (IP), or intranasal (IN). Bats were examined for lesions characteristic of white-nose syndrome (WNS) and classified as positive (P) or negative (N). Three bats identified with an asterisk died shortly after challenge and were excluded from analyses. NS=not sampled.

Bat ID No.	Age/Sex	Treatment	Route	Found dead (FD)/ Euthanized (E)	Days in hibernation	Pre-hibernation weight (g)	qPCR Ct value	WNS status by histology
1429*	AM	CAL	IM	FD	28	7.23	16.14	N
1440	AM	CAL	IM	FD	67	8.56	NS	N
1404	JM	CAL	IM	FD	58	7.69	36.74	N
1418	AM	CAL	IM	FD	Dead at study end	7.5	28.21	P
1430	AM	CAL	IM	FD	93	10.18	30.64	P
1435	AM	CAL	IM	FD	54	7.18	29.5	P
1441	AM	CAL	IM	FD	54	7.05	25.16	P
1248	AM	PBS-control	IM	FD	54	8.44	36.59	N
1434	AM	PBS-control	IM	FD	Dead at study end	8.79	20.73	P
1437	AM	PBS-control	IM	FD	67	8.82	NS	P
1409	AM	Inactivated *Pd*	IP	E	100	10.39	15.5	N
1419*	AM	Inactivated *Pd*	IP	FD	22	7.02	30.66	N
1421	AM	Inactivated *Pd*	IP	E	100	10.21	30.75	N
1436	AM	Inactivated *Pd*	IP	FD	Dead at study end	10.11	37.09	N
1403	JM	Inactivated *Pd*	IP	FD	67	8.2	NS	N
1406	JM	Inactivated *Pd*	IP	E	100	11.83	18.88	N
1414	AM	Inactivated *Pd*	IP	FD	93	9.97	33.27	P
1431	AM	Inactivated *Pd*	IP	E	100	9.67	NS	P

1405	JM	Inactivated *Pd*	IP	FD	Dead at study end	9.14	27.9	P
1249	AM	RCN-CAL/RCN-SP	IM	FD	74	8.51	No Amp	N
1413	AM	RCN-CAL/RCN-SP	IM	FD	49	8.48	33.32	N
1417	AM	RCN-CAL/RCN-SP	IM	FD	79	9.72	28.62	N
1424	AM	RCN-CAL/RCN-SP	IM	FD	77	7.66	29.82	N
1426	AM	RCN-CAL/RCN-SP	IM	FD	49	7.06	31.82	N
1427	AM	RCN-CAL/RCN-SP	IM	FD	45	6.96	34.98	N
1443*	AM	RCN-CAL/RCN-SP	IM	FD	9	7.4	23.95	N
1446	AM	RCN-CAL/RCN-SP	IM	FD	Dead at study end	10.16	NS	N
1410	JM	RCN-CAL/RCN-SP	IM	FD	49	7.27	33.47	N
1402	AM	RCN-CAL/RCN-SP	IM	E	100	10.27	NS	N
1445	AM	RCN-CAL/RCN-SP	IM	FD	57	7.09	NS	P
1411	AM	RCN-CAL	IN	FD	40	7.23	34.52	N
1425	AM	RCN-CAL	IN	FD	Dead at study end	9.73	36.12	N
1246	AM	RCN-CAL	IN	FD	45	9.41	34.73	P
1420	AM	RCN-CAL	IN	FD	Dead at study end	9.25	24.58	P
1428	AM	RCN-CAL	IN	E	100	10.15	NS	P
1447	AM	RCN-CAL	IN	FD	74	7.87	31.86	P
1449	JM	RCN-CAL	IN	E	100	10.02	32.57	P

Table S2. Data collected from *Myotis lucifigus* vaccinated with different treatments and then challenged with *Pseudogymnoascus destructans* (*Pd*) just prior to hibernation. All bats were juvenile males. Treatments included raccoon pox virus (RCN) expressing calnexin (CAL) or serine protease (SP) in combination, delivered via intramuscular (IM) injection or orally. Bats were examined for lesions characteristic of white-nose syndrome (WNS) and the number of *Pd* invasion sites were quantified. Two bats identified with an asterisk that failed to thrive after capture and died shortly after challenge were not included in analyses. ND =not determined

Bat	Treatment	Route	Cage	Found dead (FD)/ Euthanised (E)	Days in hibernation	Initial weight (g)	Carcass weight (g)	qPCR Ct score - left	qPCR Ct score -right	% wing area fluorescent under UV light	# Pd invasion sites by histology
2782	RCN-*luc* Control	IM	A	E	126	10.1	7.2	33.9	34.2	0	1
2762	RCN-*luc* Control	IM	B	E	126	10.2	7.2	29.8	30.6	0	0
2781	RCN-*luc* Control	IM	B	E	126	9.5	5.8	28.5	31.7	0	9
2789	RCN-*luc* Control	IM	B	FD	118	11.2	5.6	24.9	24.7	30-45	89
2790	RCN-*luc* Control	IM	A	FD	Dead at study end	9.7	4.6	31.2	33.5	2	11
2787	RCN-*luc* Control	oral	B	FD	69	9.5	4.4	31.8	33.7	0	1
2761	RCN-*luc* Control	oral	A	FD	112	8.8	2.5	28	31.3	10	37
2758	RCN-*luc* Control	oral	A	FD	122	11.6	5.5	30.8	34.2	5	0
2822	RCN-*luc* Control	oral	A	FD	Dead at study end	7.9	ND	30.4	29.3	0	ND
2765*	RCN-*luc* Control	oral	B	FD	26	6.3	4.6	ND	ND	0	0
2820	RCN-CAL/RCN-SP	IM	A	E	126	9.6	6.1	31.8	32.1	0	4
2801	RCN-CAL/RCN-SP	IM	A	E	126	10.3	7.2	31.9	31.4	0	0
2804	RCN-CAL/RCN-SP	IM	A	E	126	10.3	7	32	31.7	0	3
2807	RCN-CAL/RCN-SP	IM	A	E	126	10.9	7.2	31.8	32.8	0	0
2811	RCN-CAL/RCN-SP	IM	A	E	126	10.3	6.3	28.6	27.6	1	1
2818	RCN-CAL/RCN-SP	IM	B	E	126	10.4	6.2	30.5	30	0	2

2791	RCN-CAL/RCN-SP	IM	B	E	126	10.0	5.4	25.9	23.6	40	98
2808	RCN-CAL/RCN-SP	IM	B	E	126	13.3	6.0	32.5	32.8	0	2
2816	RCN-CAL/RCN-SP	IM	B	FD	119	9.3	4.9	28.9	29.6	1	13
2809	RCN-CAL/RCN-SP	IM	B	FD	122	10.0	4.9	24.7	26.8	5	45
2775	RCN-CAL/RCN-SP	oral	A	E	126	10.1	6.3	29.1	30	1	3
2794	RCN-CAL/RCN-SP	oral	A	E	126	10.3	7.2	26.9	30.3	0	1
2783	RCN-CAL/RCN-SP	oral	A	E	126	10.9	7.2	27.6	28.9	1-3	1
2752	RCN-CAL/RCN-SP	oral	A	E	126	8.5	7.2	31	38	0	5
2753	RCN-CAL/RCN-SP	oral	B	E	126	9.6	7.1	31.8	29.5	0	6
2771	RCN-CAL/RCN-SP	oral	B	E	126	10.4	7.6	27.4	26	2	55
2780	RCN-CAL/RCN-SP	oral	B	E	126	11.4	7.8	32.6	29.9	0	1
2763	RCN-CAL/RCN-SP	oral	B	FD	122	12.9	5.3	33.9	33	8	0
2779*	RCN-CAL/RCN-SP	oral	A	FD	3	6.2		ND	ND	0	0
